# Tumor Molecular Profiling for an Individualized Approach to the Treatment of Hepatocellular Carcinoma: A Patient Case Study

**DOI:** 10.3390/biomedicines6020046

**Published:** 2018-04-17

**Authors:** Kristine Posadas, Anita Ankola, Zhaohai Yang, Nelson S. Yee

**Affiliations:** 1 Pennsylvania State University College of Medicine, Hershey, PA 17033, USA; kposadas@pennstatehealth.psu.edu; 2 Department of Radiology, Penn State Health Milton S. Hershey Medical Center, Hershey, PA 17033, USA; aankola@pennstatehealth.psu.edu; 3 Department of Pathology, Penn State Health Milton S. Hershey Medical Center, Hershey, PA 17033, USA; zyang2@pennstatehealth.psu.edu; 4 Division of Hematology-Oncology, Department of Medicine, Penn State Health Milton S. Hershey Medical Center; Experimental Therapeutics Program, Penn State Cancer Institute, Pennsylvania State University College of Medicine, Hershey, PA 17033, USA

**Keywords:** hepatocellular carcinoma, immunohistochemistry, molecular profiling, next-generation sequencing, precision medicine, predictive biomarkers

## Abstract

Hepatocellular carcinoma (HCC) is increasing in incidence, and the associated mortality rate remains among the highest. For advanced HCC, sorafenib has been shown to slightly prolong survival, and regorafenib and nivolumab, both recently approved by the United States Food and Drug Administration (FDA), may produce clinical benefits to a limited extent. Systemic chemotherapy has been shown to produce a modest response, but there is no clinically valid biomarker that can be used to predict which patients may benefit. In this case study, we present two patients with metastatic HCC, they received systemic treatment using capecitabine, oxaliplatin, and either bevacizumab or sorafenib. The tumor response to treatment was determined by the progression-free survival (PFS). Molecular profiling of the tumors showed differential expression of biochemical markers and different mutational status of the *TP53* and β-catenin (*CTNNB1*) genes. We hypothesize that the PFS correlates with the tumor molecular profiles, which may be predictive of the therapeutic response to systemic chemotherapy. Further investigation is indicated to correlate tumor biomarkers and treatment responses, with the objective of personalizing the therapies for patients with advanced HCC.

## 1. Introduction

Hepatocellular carcinoma (HCC) is increasing in incidence, and the associated mortality rate remains among the highest [1]. For patients with localized HCC, surgical resection and liver transplantation may be offered with curative intent. Palliative local therapy, such as chemoembolization, radiofrequency ablation, and stereotactic body radiation therapy, are options for treatment [2]. However, for patients with advanced or metastatic HCC, palliative systemic treatment is the only option, and the associated survival benefit is limited [3].

For select patients with advanced HCC, sorafenib is the standard first-line systemic treatment [4]. In the second-line setting, regorafenib (a small molecule inhibitor targeting tyrosine kinases and angiogenesis receptors) and nivolumab (an anti-PD-1 monoclonal antibody) have been recently approved by the United States Food and Drug Administration (FDA) for patients with advanced HCC, which have failed to respond to sorafenib [5,6]. According to the National Comprehensive Cancer Network (NCCN), patients with unresectable HCC should receive systemic chemotherapy preferably in a clinical trial setting [7]. HCC is resistant to conventional chemotherapeutic agents possibly related to pathogenic mutations in certain genes such as *TP53* and *CTNNB1* (coding for β-catenin), which are commonly mutated in HCC [3]. This is supported by evidence that mutated TP53 and CTNNB1 contribute to increased cell proliferation and reduced apoptosis in HCC [8,9]. Multiple studies have indicated that patients with HCC that carry mutations in *TP53* have a relatively poor prognosis [10]. Molecular profiling of HCC has been performed to characterize this type of malignancy with the hope of identifying predictive biomarkers and therapeutic targets.

A retrospective study showed considerable molecular heterogeneity among 350 specimens of HCC [11]. An immunohistochemical analysis indicated various frequencies of change in the expression of protein biomarkers and the associated potential therapeutic agents. For instance, a decreased expression of thymidine synthetase (TS) and excision repair cross-complementation group 1 (ERCC1) was found in 79.8% and 66.1% of specimens, respectively. Reduced expression of these biochemical markers suggests potential benefits from fluoropyrimidines and platinum agents, respectively [12,13]. Furthermore, genetic mutations were most frequently identified in the *TP53* and *CTNNB1* genes in 30% and 20% of the tested specimens, respectively. While there was no standard effective chemotherapy for advanced HCC, early phase studies suggested capecitabine and oxaliplatin (CAPOX) to be effective in patients with HCC. This was demonstrated in a phase II study that showed modest anti-tumor activity when using CAPOX as a first-line therapy, with a median progression-free survival (PFS) of 4.1 months [14]. Furthermore, a clinical trial to investigate a combination of capecitabine, oxaliplatin, and bevacizumab (CAPOX-B) as a first-line regimen in patients with advanced/metastatic HCC showed a median PFS of 6.8 months [15].

In this case study, we present two patients who were diagnosed with metastatic HCC, analyzed for tumor molecular profiles, and treated with CAPOX in combination with either bevacizumab or sorafenib. These two patients had different clinical features regarding the etiology of HCC and treatment responses, and the molecular profiles of their tumors were distinct in the expression of biochemical markers and genomic DNA mutations. The correlation of the therapeutic response with the tumor molecular profiling suggests the potential for developing predictive biomarkers using a large data set, and evaluating the use of individualized treatment for patients with HCC in future prospective studies.

## 2. Case Reports

### 2.1. Patient #1

This is a 65-year-old Caucasian man who presented with progressive weakness and paresthesia in the bilateral lower extremities. He also complained about urinary retention lasting two days. His past medical problems included hypertension, psoriatic arthritis, lymphedema in the right lower extremity, a cardiac murmur, and osteoarthritis in bilateral knees status post left knee replacement. He had no history of hepatic cirrhosis, diabetes mellitus, steatohepatitis, viral hepatitis, alcoholism, hemochromatosis, or Wilson’s disease. His family history showed significant “liver cancer” and hemochromatosis in his father and “cancer in a digestive organ” in his paternal grandmother. He had worked in the navy as a maintenance supervisor and a shuttle bus driver. He had previously smoked one pack of cigarettes per day for 20 years and quit smoking 20 years ago; he had previously consumed alcohol and he last drank in December 2015. The physical examination was remarkable for chronic edema in the bilateral lower extremities, the spine was non-tender, and no peritoneal ascites was noted. Laboratory tests showed elevated serum alpha-fetoprotein (AFP) level in the liver, corresponding to 60.9 ng/mL (reference range: 0–15 ng/mL), and normal AFP-L3 (3.6%, reference range: 0–9.9%), while his carbohydrate antigen 19–9, carcinoembryonic antigen, prostate-specific antigen, and β-human chorionic gonadotropin were all within normal limits. Viral hepatitis serology was non-reactive for both the hepatitis B viral envelope antigen and the hepatitis C virus.

**Figure 1:**
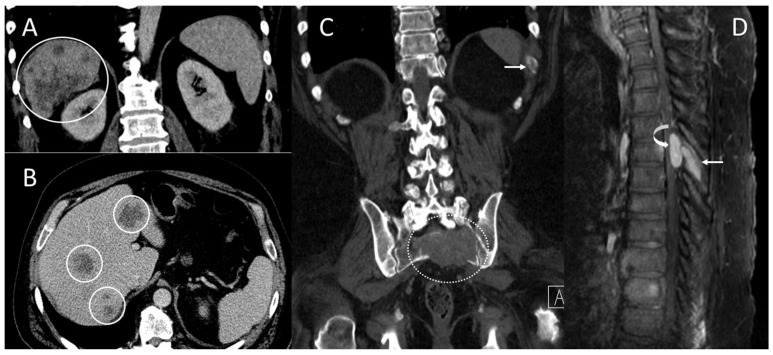
Computed tomography (CT) scans and magnetic resonance imaging (MRI) scans at initial diagnosis showed metastatic lesions in the liver, left 11th rib, 6th thoracic spine (T6), and sacrum. (**A**) Coronal and (**B**) axial contrast-enhanced CT scans demonstrate multifocal heterogeneous irregular hepatic tumors of varying sizes throughout the liver (solid circles) primarily in the right hepatic lobe. (**C**) Coronal CT scans in bone window demonstrate a lytic bone metastasis involving the left eleventh rib (arrow) and a large, destructive upper sacral metastasis (dashed circle). (**D**) Sagittal contrast-enhanced MRI scans demonstrate an enhancing mid-thoracic spine epidural metastasis (curved arrow) with an adjacent bone metastasis involving the T6 spinous process (arrow).

**Figure 2:**
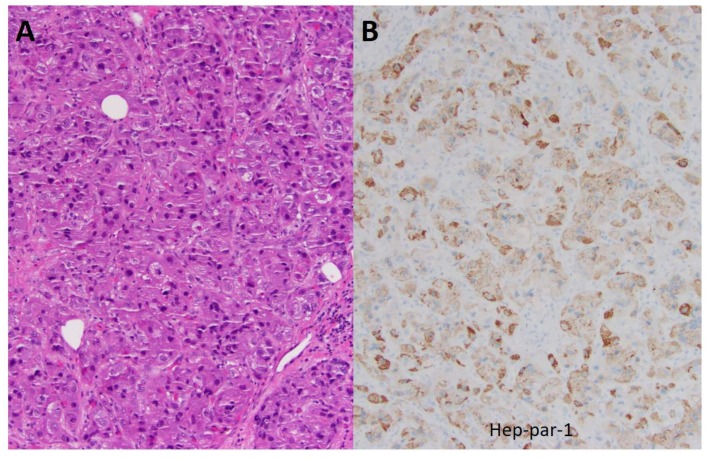
Histopathological and immunohistochemical analysis of the biopsied T6 epidural mass. (**A**) The hematoxylin- and eosin-stained section shows infiltration by nests of polygonal cells with enlarged atypical nuclei and abundant eosinophilic cytoplasm. Nuclear pseudo-inclusion and mitotic figures are apparent. (**B**) Immunohistochemical analysis for Hep-Par-1 shows granular cytoplasmic staining, supporting a diagnosis of metastatic hepatocellular carcinoma (original magnification ×200).

The initial evaluation conducted using computed tomography (CT) scans (December 2015) revealed multiple lesions in the liver and bones (T6 spine, sacrum, bilateral ribs) (Figure 1). He underwent a T5–T7 laminectomy, an open biopsy of the thoracic intraspinal (extradural) lesion, and excision of the thoracic epidural neoplasm. The pathology of the biopsied T6 epidural mass showed metastatic carcinoma, and the histopathology and immunohistochemical staining for Hep-Par-1 were consistent with a hepatic primary tumor (Figure 2). Thus, this patient had stage IV B (T3a N0 M1) HCC. Starting in February 2016, he was started on sorafenib (200 mg orally every 12 h daily) as a first-line therapy. Two months later, CT scans showed enlarged tumors in the liver, with omental and mesenteric carcinomatosis, stable osseous metastases, and the serum AFP-liver level increased to 71 ng/mL, consistent with tumor progression.

In May 2016, he started immunotherapy on a clinical trial using an investigative anti-PD-1 monoclonal antibody. The patient received four cycles of this treatment (a total of eight weeks), and then the tumor progressed. This was evidenced by CT scans (July 2016) showing an increase in both size and number of omental, peritoneal, mesenteric metastases, a stable disease in liver, bones, and lymph nodes, and the serum AFP-liver level increased further to 102.3 ng/mL.

Molecular profiling of the epidural metastatic tumor was performed by the Caris^®^ Life Sciences (https://www.carislifesciences.com/cmi-overview/). The materials and methods for the tumor molecular profiling were previously described in detail [11]. Briefly, using formalin-fixed paraffin-embedded tumor tissues, successive 4 μm sections were generated until sufficient material for testing was obtained. For the molecular analysis, tumor cells were excised by microdissection, until a total area of at least 50 mm^2^ was obtained. The expression of a panel of protein and RNA biomarkers predictive of the response to cytotoxic chemotherapy and molecularly targeted agents was determined by immunohistochemistry (IHC), chromogenic in-situ hybridization (CISH), and RNA sequencing (RNA-Seq). The expression levels of various biochemical markers and the associated therapeutic agents with potential benefits are listed in Table 1. Notably, the immunohistochemical analysis of tumor tissues showed a lack of expression for thymidine synthetase (TS) and a diminished expression of excision repair cross-complementation group 1 (ERCC1), suggesting a potential benefit from capecitabine and oxaliplatin, respectively. Moreover, next-generation sequencing (NGS) of tumor genomic DNA showed no pathogenic mutation in the tested genes. In particular, no pathogenic mutation was detected in the genes *TP53*, *CTNNB1*, *BRCA1*, *BRCA2*, *BRAF*, *KRAS*, *EGFR*, and *PIK3CA* (Table 2).

**Figure 3:**
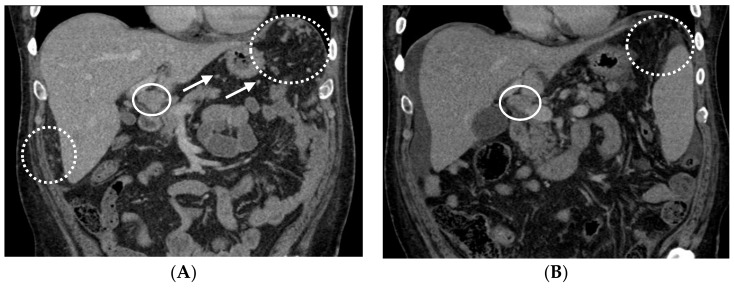
Following cycle 3 and cycle 6 with capecitabine, oxaliplatin, and bevacizumab (CAPOX-B), the CT scans showed tumor response. (**A**) Following cycle 3 with CAPOX-B, the coronal contrast-enhanced CT scans demonstrate innumerable left sub-diaphragmatic and peri-hepatic omental nodules (dashed circles) along with peri-gastric nodules (several annotated by arrows) consistent with omental carcinomatosis and mesenteric metastases. Peri-portal lymphadenopathy can be observed in the solid circle. (**B**) Following cycle 6 with CAPOX-B, the coronal contrast-enhanced CT scans demonstrate a decrease in omental metastases (dashed circle) and peri-portal lymphadenopathy (solid circle), and a small amount of peri-hepatic ascites.

On the basis of the tumor molecular profile, treatment with capecitabine, oxaliplatin, and bevacizumab (CAPOX-B) was initiated. The selection of this regimen (CAPOX-B) was based on results of the phase II study in patients with advanced HCC. The combination appeared efficacious and tolerable. This study demonstrated a median PFS of 6.8 months, a median overall survival (OS) of 9.8 months, a partial response rate of 20%, and a disease control rate of 77.5% [15]. For every 21-day cycle of the CAPOX-B regimen, capecitabine (825 mg/m^2^) was administered orally twice daily on day 1 through to day 14, oxaliplatin (130 mg/m^2^) was administered intravenously on day 1, and bevacizumab (5 mg/kg) was administered intravenously on day 1. Following treatment with three cycles of CAPOX-B, CT scans revealed a decrease in the size of the hepatic and omental lesions with stable osseous and peri-portal lymphadenopathy (Figure 3A). After another three cycles of therapy, CT scans showed a continued decrease in the size of the hepatic, lymph nodal, and omental lesions (Figure 3B).

**Figure 4:**
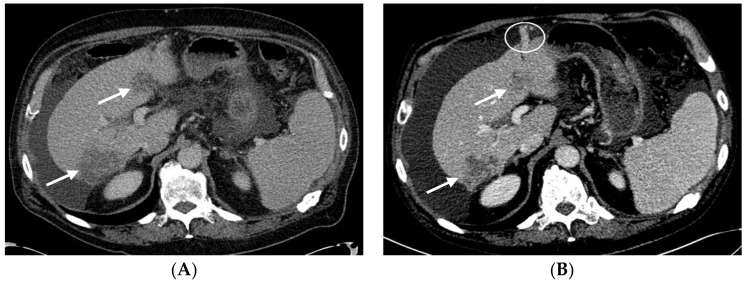
The tumors remained stable following cycle 9 and cycle 15 with CAPOX-B. (**A**) Following cycle 9 with CAPOX-B, the axial contrast-enhanced CT scans demonstrate stable heterogeneous hepatic tumors (arrows) compatible with a diagnosis of hepatocellular carcinoma. The right hepatic lobe tumor is subcapsular in location. There are small peri-hepatic and peri-gastric ascites. (**B**) Following cycle 15 with CAPOX-B, the axial contrast-enhanced CT scans demonstrate persistent heterogeneously enhanced hepatic tumors of a stable size (arrows). Progressive peri-hepatic and peri-gastric ascites with recanalized para-umbilical vein (circle) and splenomegaly can be seen.

**Figure 5:**
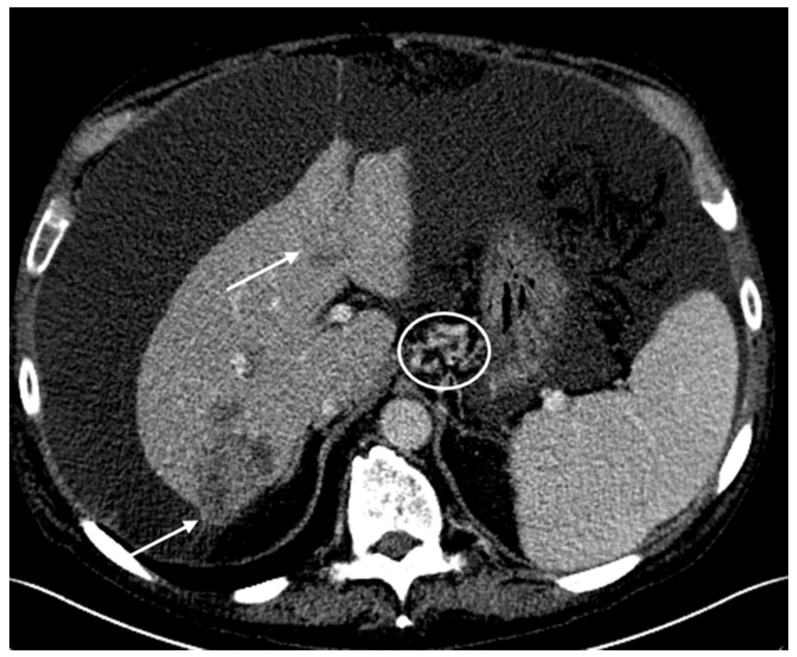
Following cycle 18 with CAPOX-B, there was evidence of tumor progression in the CT scans on the basis of the Response Evaluation Criteria in Solid Tumors (RECIST) guideline version 1.1. Axial contrast-enhanced CT scans demonstrate enlarging heterogeneous hepatic tumors (arrows). The larger right hepatic tumor now measures 6.3 cm with an exophytic component. There is a large-volume abdominal ascites that has progressively increased in size, with gastrohepatic varices (circle).

Following cycle 9 with CAPOX-B, the CT scans showed stable hepatic and metastatic lesions (Figure 4A). The hepatic and metastatic lesions remained unchanged following cycle 15 with CAPOX-B, as shown in the CT scans (Figure 4B). The patient went on to receive a total of 18 cycles of CAPOX-B; the disease eventually progressed as evidenced by new and enlarged hepatic masses in the CT scans based on Response Evaluation Criteria in Solid Tumors (RECIST) guideline version 1.1 (Figure 5). Clinically, the patient experienced fatigue, ascites, and edema in the bilateral lower extremities, all grade 3 according to the Common Terminology Criteria for Adverse Events (CTCAE Version 4.0). At that time, the patient decided to pursue the option of home hospice and he subsequently expired at home.

### 2.2. Patient #2

In contrast to patient #1, another patient with recurrent metastatic HCC was treated using a similar regimen of chemotherapy; he had a relatively short PFS and a distinct tumor molecular profile. Patient #2 is a 57-year-old Caucasian man who underwent an orthotopic liver transplantation (in April 2015) because of HCC, in the setting of a hepatitis C viral infection and hepatic cirrhosis. A pathological examination of the native liver revealed multiple foci of moderately differentiated HCC, stage pT3b, pN1, and the portal vein margin was also affected by invasive carcinoma. The past medical history was significant for hepatitis C viral infection (genotype 1; previously treated with sofosbuvir and ribavirin), hepatic cirrhosis, hypertension, aortic stenosis, and insulin-dependent diabetes mellitus. The family history was significant for “liver cancer” in his brother, colon cancer in his brother, thyroid cancer in his sister, and “cancer of type unknown to patient” in his brother. He had worked as a machine operator. He had previously smoked one pack of cigarettes daily for 30 years and he admitted to previous consumption of alcohol and intravenous drug use. At clinical presentation, he complained of fatigue and incisional pain, but denied suffering from nausea, vomiting, and diarrhea. The physical examination was remarkable for a mildly distended abdomen and chronic 1+ pitting edema in the bilateral lower extremities; there was no scleral icterus or jaundice. The serum AFP-liver level was normal at 2.1 ng/mL, and AFP-L3 was elevated at 39.1%. Surveillance CT scans (in January 2015) showed a new small thrombus within the right portal vein.

Between May and December 2015, this patient received eight cycles of adjuvant doxorubicin and sorafenib. For every 21-day cycle, doxorubicin (60 mg/m^2^) was administered intravenously on day 1, and two tablets of sorafenib (200 mg) were administered orally every 12 h continuously [16]. In December 2015, surveillance CT scans showed a new 2 cm × 1.4 cm mass in the lower lobe of the left lung and an enlarged and enhanced (tumor) thrombus within the portal vein. The biopsy of the pulmonary mass revealed metastatic carcinoma. He received stereotactic body radiation therapy (SBRT) to the tumor thrombus in the portal vein (in February 2016) and to the tumor in the left lung lobe (in March 2016). CT scans in April 2016 showed an enlarged left lower lung mass and new bilateral adrenal nodules corresponding to metastases. The serum AFP-liver level and AFP-L3 were both elevated at 20.4 ng/mL and 67.4%, respectively.

A tumor molecular profiling of the metastatic carcinoma biopsied from the left lower lobe of the lung was performed using formalin-fixed paraffin-embedded tumor tissues by Caris^®^ Life Sciences (https://www.carislifesciences.com/cmi-overview/). The results of the analyses by IHC, CISH, and RNA-Seq revealed the expression levels of biomarkers and the associated chemotherapy agents with potential benefits (Table 3). There was a decreased expression of ERCC1, suggesting the potential benefits of oxaliplatin. However, TS levels were increased, predicting a potential lack of benefits of capecitabine. The mutational analysis of genomic DNA by NGS was significant for pathogenic mutations in both *TP53* (exon 6, Y220C) and *CTNNB1* (exon 3, S37Y); there was no detected pathogenic mutation in the other genes tested.

In April 2016, the patient was started on a treatment plan using a combination of sorafenib, oxaliplatin, and capecitabine (SECOX) [17]. This regimen was previously shown to produce anti-tumor activity and was tolerable, with no treatment-related death being reported. In this single-arm, multi-center, phase II study, 51 patients with advanced HCC were enrolled and treated with the SECOX regimen. The best response rate was 16% (all partial response), the median PFS was 5.26 months, and the median OS was 11.73 months [18]. For every 14-day cycle of the SECOX regimen, two tablets of sorafenib (200 mg) were administered orally every 12 h continuously, two tablets of capecitabine (500 mg) were administered orally every 12 h on day 1 through to day 7, and 85 mg/m^2^ of oxaliplatin was administered intravenously on day 1.

Following cycle 4 of SECOX, the patient tolerated the treatment well without specific complaints. CT scans in June 2016 showed a mixed response including an interval progression of the infiltrative disease within the liver, slightly enlarged bilateral adrenals nodules, an enlarged small right upper lung lobe, and a reduction in size of the left lower lung lobe. The serum AFP-liver level and AFP-L3 were both increased, being 55 ng/mL and 55.2%, respectively. The patient received a further four cycles of SECOX with a slightly increased dosage of oxaliplatin (having been previously reduced because of thrombocytopenia). In August 2016, CT scans showed progression of the disease in the right upper lung lobe, liver, adrenals, and peritoneum on the basis of the RECIST guideline v1.1. The serum AFP-liver level and AFP-L3 both rose further to 78.3 ng/mL and 60.9%, respectively. SECOX was subsequently discontinued. PFS was 3.7 months. At that time, the patient experienced grade 1 nausea/emesis/diarrhea and transient grade 1 oxaliplatin-induced cold hypersensitivity (CTCAE v4.0). Prior to starting regorafenib for treatment, he expired at home.

### 2.3. Timeline

**Figure 6:**
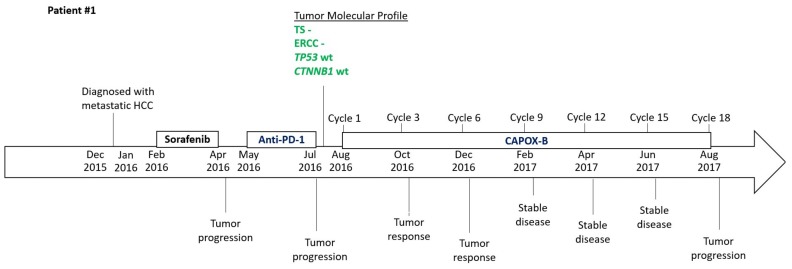

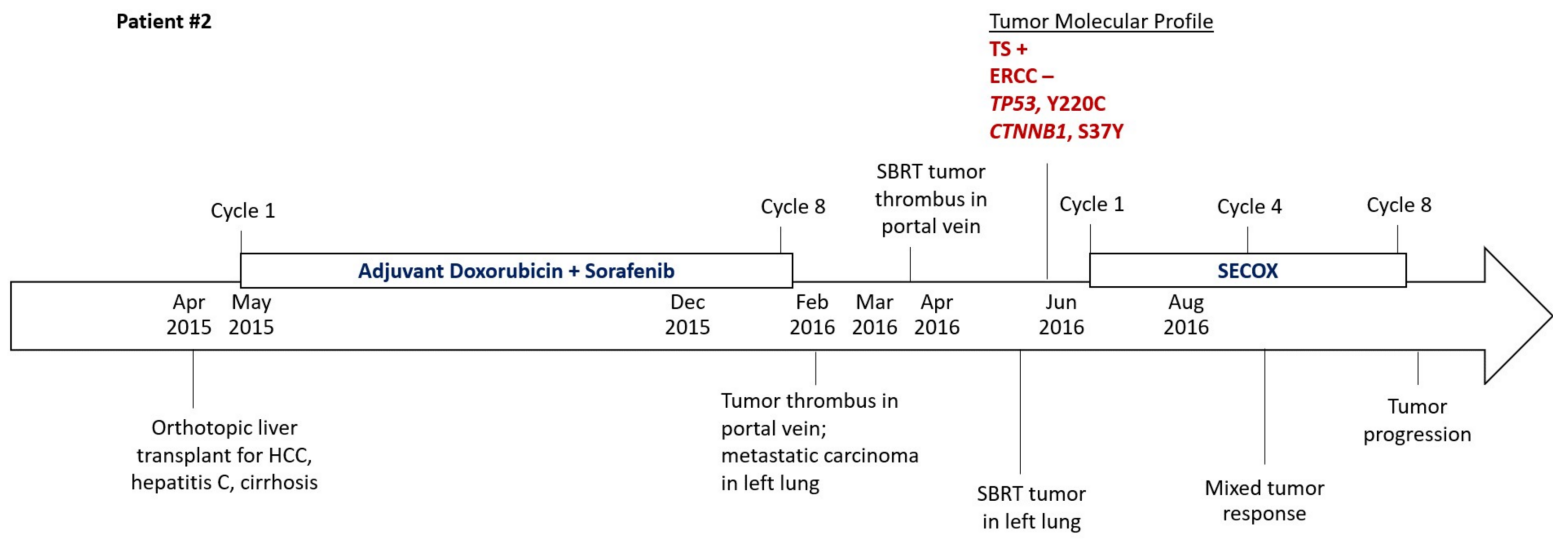
Timeline to illustrate the chronology of treatment and responses to treatment for patients #1 and #2 and the key data of their tumor molecular profiles.

The clinical data of both patients #1 and #2, including diagnosis, treatment, the key result of tumor molecular profiles, and responses to treatment, are summarized as a timeline as illustrated in Figure 6.

## 3. Discussion

In this case study, we present two patients who had different clinical features regarding the etiology of HCC and treatment responses. The molecular profiling of their tumors were distinct in the expression of biochemical markers and genomic DNA mutations. In particular, patient #1 showed an unusually good tumor response with CAPOX-B, and his tumor molecular profiling indicated a negative expression of TS and ERCC1, as well as a lack of pathogenic mutations in the *TP53* and *CTNNB1* genes. In contrast, patient #2 received eight cycles of SECOX with a PFS of 3.7 months, and his tumor molecular profiling showed negative expression of ERCC1, but increased expression of TS, and also pathogenic mutations in both the *TP53* and the *CTNNB1* genes. It is known that *TP53* and *CTNNB1* are the two most common mutations in HCC and they contribute to tumor resistance to chemotherapeutic agents. These data suggest that the tumor molecular profile of HCC may correlate with the treatment efficacy as determined by PFS.

HCC is generally considered resistant to cytotoxic chemotherapeutic drugs, but treatment responses of tumors are variable among patients. Currently, there is no predictive biomarker of treatment response for patients with HCC. These cases highlight the value of molecular profiling in HCC, especially in patients with advanced and/or metastatic HCC that has progressed following standard therapies. Patient #1 had a PFS of 12.3 months; this is well beyond the median PFS of 6.8 months as reported in the phase II trials using CAPOX or nivolumab [6,14], and almost twice as long as the median PFS in the study evaluating CAPOX-B as a first-line treatment [15]. Given the patient’s negative IHC analysis for TS and ERCC1 and the lack of pathogenic mutations in the tested genes, we hypothesize that his robust response to CAPOX-B was related to a favorable molecular profile. His response to CAPOX-B was particularly impressive, considering his PFS in response to CAPOX-B as a third-line treatment. On the other hand, patient #2 had a relatively short PFS of 3.7 months, less than the median PFS of 5.26 months as reported in the phase II study evaluating the efficacy of SECOX in advanced HCC [18]. Similarly, we hypothesize that patient #2’s low response to SECOX was related to his molecular profile which showed a potential lack of benefit from capecitabine and exhibited pathogenic mutations in two key genes, *TP53* and *CTNNB1*. No data on tumor molecular profiling were available from the previous phase II studies that investigated CAPOX-B and SECOX.

How the etiology of HCC contributes to therapeutic responsiveness or resistance is unclear, though the various modalities of treatment for patients with HCC have been the same in clinical practice regardless of their etiology. While the etiology of patient #1’s HCC is unclear, patient #2 undoubtedly had developed HCC as a consequence of a hepatitis C viral infection and hepatic cirrhosis. It is noteworthy that the tumor profile of patient #1 showed negative TS expression and no pathogenic mutation in *TP53*, and that of patient #2 showed positive TS expression with pathogenic mutation in *TP53*; such an association is consistent with the data of a previous study [11]. Nevertheless, it will be important to determine any association between the etiology of HCC and the tumor molecular profiles, as well as the clinical efficacy of systemic treatments including chemotherapy.

## 4. Conclusions and Future Perspectives

This case study sheds new light on the potential value of molecular profiling of advanced/metastatic HCC in terms of guiding treatment. However, definitive conclusions on the clinical utility of the tumor molecular profiles of HCC as predictive biomarkers cannot be drawn based on the limited scope of the data from these case reports. Retrospective studies using data from a large patient population are indicated to correlate biomarkers with treatment responses. Ultimately, a prospective clinical study will be needed to test the hypothesis that molecular profile-based biomarkers can predict clinical outcomes. Our goal is to identify and develop predictive biomarkers for treatment response in order to help guide the selection of personalized therapy for patients with HCC.

## Figures and Tables

**Table 1:** Biomarker analysis and associated therapies.

Test	Method	Result	Value	Conditions for a Positive Results	Potential Benefit	Therapies
TS	IHC	Negative	0 + 100%	≥1 + ≥10%	Yes	capecitabine, fluorouracil, pemetrexed
ERCC1	IHC	Negative	2 + 5%	≥3 + ≥10% or ≥2 + ≥50%	Yes	carboplatin, cisplatin, oxaliplatin
TUBB3	IHC	Negative	0 + 100%	≥2 + ≥30%	Yes	docetaxel, *nab*-paclitaxel, paclitaxel
TOP2A TOPO1	IHC IHC	Negative Positive	2 + 3% 2 + 50%	≥1 + ≥10% ≥2 + ≥30%	No Yes	doxorubicin, epirubicin, liposomal doxorubicin irinotecan, topotecan
HER2/Neu	CISH, IHC, NGS	Not amplified, Negative			No	adotrastuzumab emtansine (T-DM1), pertuzumab, trastuzumab, lapatinib
ALK	RNA-Seq	Fusion not detected			No	ceritinib, crizotinib
ROS1	RNA-Seq	Fusion not detected			No	crizotinib

The immunohistochemical analyses were developed by Caris MPI, Inc. d/b/a Caris Life Sciences^®^, which also determine their performance characteristics. The therapies with potential benefits are based on the body of evidence, overall clinical utility, competing biomarker interactions, and tumor type from which the evidence was gathered, as listed in www.carislifesciences.com. The value represents the staining intensity and the percent of cells showing staining. ALK: anaplastic lymphoma kinase. CISH: chromogenic in-situ hybridization. ERCC1: excision repair cross-complementation group 1. HER2/Neu: human epidermal growth factor receptor 2. IHC: immunohistochemistry. RNA-Seq: RNA sequencing. ROS1: avian UR2 sarcoma virus oncogene. TOP2A: DNA topoisomerase II alpha. TOPO1: DNA topoisomerase I. TS: thymidine synthetase. TUBB3: tubulin beta 3 class III.

**Table 2:** Tumor genomic DNA analysis by next-generation sequencing for genetic mutations.

ABL1	BRCA2	EGFR	HRAS	NF1	RET
AKT1	c-KIT	HER2/Neu (ERBB2)	IDH1	NOTCH1	ROS1
ALK	CDK4	ERBB3	IDH2	NRAS	SMO
Androgen Receptor	CDKN2A	FGFR1	JAK2	NTRK1	SRC
APC	CHEK1	FGFR2	KDR (VEGFR2)	PDGFRA	TP53
ARAF	CHEK2	FGFR3	KRAS	PDGFRB	VHL
ATM	cMET	FLT3	MEK1	PIK3CA	WT1
BAP1	CSF1R	GNA11	MEK2	PTCH1	
BRAF	CTNNB1	GNAQ	MLH1	PTEN	
BRCA1	DDR2	GNAS	MPL	RAF1	

Using the Illumina NextSeq platform, a direct sequence analysis was performed on the genomic DNA isolated from a formalin-fixed paraffin-embedded tumor sample. This analysis can detect all variants with >99% confidence based on the mutational frequency present as well as the amplicon coverage. This test is sensitive enough to detect as little as a 10% population of cells containing a genomic mutation. The details can be found at www.carislifesciences.com.

**Table 3:** Biomarker analysis and associated therapies.

Test	Method	Result	Value	Potential Benefit	Therapies
TS ERCC1	IHC IHC	Positive Negative	1 + 10% 2 + 35%	No Yes	capecitabine, fluorouracil, pemetrexed carboplatin, cisplatin, oxaliplatin
TUBB3	IHC	Negative	1 + 90%	Yes	docetaxel, nab-paclitaxel, paclitaxel
TOP2A	IHC	Positive	2 + 20%	Yes	doxorubicin, epirubicin, liposomal doxorubicin
TOPO1	IHC	Positive	2 + 80%	Yes	irinotecan, topotecan

Value represents staining intensity and percent of cells showing staining. ERCC1: excision repair cross-complementation group 1. IHC: immunohistochemistry. TOPO1: DNA topoisomerase I. TOP2A: DNA topoisomerase II alpha. TS: thymidine synthase. TUBB3: tubulin beta class III.
